# Wnt Signaling Relies on Nuclear Armadillo

**DOI:** 10.1371/journal.pbio.0020102

**Published:** 2004-02-10

A couple of years ago, a paper was published in a high-profile journal that challenged a long-established model of cell signaling. While researchers in the field mostly greeted the results with skepticism, some went into the lab to investigate the discrepancy. Many elements of this pathway, called the Wnt pathway, have been well characterized. The standard model of Wnt signaling holds that when the Wnt protein binds to its receptor, it initiates a labyrinthine signaling cascade that sends a protein called β-catenin into the cell's nucleus where, together with a protein complex, it initiates transcription. In the absence of this signal, β-catenin binds to an inactivating complex in the cytoplasm and is targeted for degradation. The paper that disputed this view suggested that β-catenin can effect gene expression without entering the nucleus and that it can activate the Wnt pathway while tethered to the cell membrane.

Before that paper was published, Nicholas Tolwinski and Eric Wieschaus had shown that β-catenin, also known as Armadillo (Arm) in the fruitfly, is sent into the nucleus in response to Wnt signaling. Upon entering the nucleus, Arm interacts with a second protein complex to activate transcription. Now Tolwinski and Wieschaus have reexamined the function of Arm in the fruitfly and have demonstrated that the pathway “in fact does depend on the nuclear localization of β-catenin.” While their paper was in the final stages of acceptance, the dissenting paper was retracted, after it was learned that the results had been fabricated. Tolwinski and Wieschaus' findings confirm what had already been known about Arm's role in Wnt signaling and also fill in important details about how it works.

Multicellular organisms rely on elaborate communication networks of signaling proteins and enzymes to exchange information between cells. The Wnt signaling pathway regulates the expression of a host of different genes during embryogenesis to control body patterning and cell differentiation in organisms from fruitflies to mammals. Miscommunications in this tightly regulated pathway contribute to a variety of developmental defects and cancers.

In the developing fruitfly, Wnt signaling is normally restricted to the front of each larval segment, where it produces a smooth surface; the rear of the segments, where Wnt signaling is absent, is hairy. If Wnt signaling is overexpressed, it produces fruitfly larvae with only smooth segments; lack of Wnt signaling produces hairy segments. Using the smooth phenotype as a measure of Wnt signaling, Tolwinski and Wieschaus delved deeper into the role of Arm in this signaling process.

These experiments are complicated because Arm functions not just in Wnt signaling, but also in cell adhesion. The trick is to make the endogenous Arm (the version encoded by the fly genome) defective for signaling, while leaving the cell adhesion functions fairly normal. Set against this “background,” an additional Arm protein is expressed that is tethered to the membrane; it still retains the protein domains required for signaling, but it's stuck on the inside of the membrane and can't move into the nucleus. In this background, any signaling in response to Wnt must mean that Arm can signal to the nucleus without actually having to get inside. Tolwinski and Wieschaus prove—again—that this is not the case. They do this by showing that the weak, medium, and strong endogenous Arm mutants—these classifications reflect the severity of the mutations' effects—have different effects on signaling in the presence of the membrane-tethered Arm.

It's clear, they argue, that tethered Arm cannot signal on its own and must somehow be helping the weaker mutants signal. To further investigate how the tethered Arm activates the endogenous mutants, Tolwinski and Wieschaus developed two new and “cleaner” Arm mutants that impair Arm's signaling ability but have no effect on its cell adhesion function. The tethered Arm could not produce a completely smooth phenotype with these nonsignaling endogenous mutants. These experiments, the authors conclude, indicate that the tethered form of Arm produces its transcriptional effects by activating the endogenous Arm protein. Normal activation of the pathway liberates Arm proteins from the inactivating complex, which allows them to enter the nucleus and activate transcription. Tethered Arm appears to accomplish this by sequestering the inactivating complex at the cell membrane, preventing it from interfering with endogenous Arm.

Even though Tolwinski and Wieschaus started these experiments based on what turned out to be fabricated results, their investigations produced valuable contributions. They not only reaffirm the standard model of Wnt signaling, but reveal important new insights into the workings of a major player in the pathway.

